# Sociodemographic Factors and Determinants of Mental Health in the African American Population A Cross-Sectional Study

**DOI:** 10.3390/healthcare14060700

**Published:** 2026-03-10

**Authors:** Yesenia Acosta-Vinueza, Rodrigo Alvear-Reascos

**Affiliations:** Faculty of Health Sciences, School of Medicine, Universidad Técnica del Norte, Ibarra 100105, Ecuador

**Keywords:** mental health, prevalence, social determinants of health, mental disorders, Afro-descendant population

## Abstract

**Background/Objectives:** Mental disorders are a growing public health concern in Latin America, particularly among marginalized populations. This study aimed to identify the prevalence and associated risk factors of mental disorders in Afro-descendant communities of the Río Chota Basin, a historically excluded population facing persistent social and economic inequalities. **Methods:** A cross-sectional descriptive study was conducted in several rural communities using random sampling. A total of 557 participants were assessed for sociodemographic factors and mental health status using the following validated instruments: the Global Mental Health Assessment Tool–Primary Care (GMHAT/PC), the International Physical Activity Questionnaire (IPAQ), and the WHO STEPS questionnaire. Data was analyzed using descriptive statistics and inferential tests to determine associations between psychosocial, behavioral, and socioeconomic variables and the presence of mental disorders. **Results:** The overall prevalence of mental disorders was extremely high (60.7%), exceeding national and regional estimates. The most prevalent conditions were major depressive disorder (15.6–17.9%), anxiety disorders (10.2–12.3%), and psychosis with depressive symptoms (8.3–11.5%), with higher rates among women. Low fruit and vegetable intake and income below the minimum wage were significantly associated with greater prevalence. Severe stress and experiences of abuse or maltreatment showed the strongest associations with mental disorders (*p* < 0.001). **Conclusions:** Afro-descendant communities in the Río Chota Basin experience a disproportionately high burden of mental illness influenced by intertwined socioeconomic, psychosocial, and behavioral determinants. Culturally sensitive interventions that promote resilience, community support, and reduction in structural inequalities are urgently needed.

## 1. Introduction

The history of the African American community in Latin America is deeply marked by the transatlantic slave trade, which is considered the largest forced migration in history [1]. More than 500 years ago, African Americans arrived in the region under adverse conditions driven by racist, economic, and religious motives. Many slaves were brought to serve as warriors, others as labor for plantations, mines, domestic services, and, to a lesser extent, as soldiers. The struggle for freedom—initiated through failed escape attempts, confrontations, and claims—finally bore fruit in the early 19th century. However, after the progressive abolition of slavery, African American settlement groups have continued to be viewed and treated as a danger to social security, leading to exclusion and racial violence.

Over the last 250 years, these groups have rebuilt their identity and sense of belonging in their new territories, yet they still face poverty, social and economic hardships, and limited access to healthcare. Geographic isolation, while favoring the preservation of identity, culture, and social cohesion, has also led to endogamy, predisposing them to the expression of genetic, cardiovascular, and mental diseases [2].

Various international studies have shown that a history of violence, trauma, deprivation, racism, and post-migration difficulties is associated with high rates of mental disorders—mainly post-traumatic stress disorder and depression among Americans of African descent and African migrant populations in Europe, Asia, and the Americas. Social and economic determinants make a significant contribution to the development of problems such as depression, anxiety, and substance use disorders. Additionally, exposure to natural disasters, geographic isolation, illiteracy, medical comorbidities, and low income markedly affects the quality of life of these communities [3,4,5,6,7].

Mental health problems in migrants and refugees are reflected in difficulties coping with stress, working productively, and contributing to society due to various barriers. Likewise, violent traumas and severe deprivation can explain a heightened risk of mental illnesses [2]. A study conducted with 500 low-income African Americans and Latinos highlights the harmful consequences of cumulative stress and trauma on mental health [8]. Unresolved traumas and emotional patterns are passed down through generations, influencing current family dynamics [9]. It is well-known how an adverse environment can provoke genetic polymorphisms under conditions of social stress and economic hardship, emphasizing the importance of the interaction between environmental and genetic factors in the manifestation of mental disorders [10].

In Ecuador, most African American settlements that arrived as slaves are in rural riverside areas in the north of the country, where vulnerability to disasters and social exclusion is especially pronounced. However, despite the magnitude and specificity of these challenges, there is a notable lack of published studies in Ecuador that systematically address the mental health of Afro-descendant communities. The scarce existing literature is focused on demographic or cultural aspects, neglecting a thorough analysis of psychosocial factors as determinants of the prevalence of mental disorders within these groups [11,12].

The lack of local research hampers the understanding of an empirically observed problem and consequently limits the design of interventions and the development of policies aimed at optimally and pertinently addressing this situation. Therefore, it is essential to obtain scientific information regarding the interaction of social and economic factors on the mental health of these groups [13,14,15].

**Figure 1:**
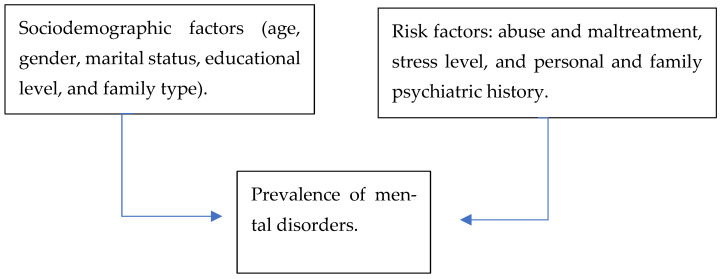
Conceptual framework of the study showing sociodemographic factors (age, gender, marital status, educational level, and family type) and risk factors (abuse and maltreatment, stress level, and personal and family psychiatric history) potentially associated with the prevalence of mental disorders.

This study aimed to estimate the prevalence of mental disorders and identify their social and economic determinants among adults from African American communities in the Chota River basin, Ecuador. Specifically, associations were evaluated between mental disorders diagnosed via GMHAT/PC and factors such as perceived stress, experiences of abuse, socioeconomic level, and medical comorbidities, using two-stage cluster probability sampling (n = 557). The findings will provide local evidence to guide culturally appropriate preventive interventions. [16,17]. The conceptual framework of the study and the main groups of variables evaluated are summarized in Figure 1.

**Research** **Hypothesis** **1** **(H1):** *There is a significant association between sociodemographic factors (age, gender, marital status, educational level, and family type) and the prevalence of mental disorders.*

**Research** **Hypothesis** **2** **(H2):** *Risk factors (abuse and maltreatment, stress levels, and personal and family psychiatric history) are associated with a higher prevalence of mental disorders.*

**Research** **Question:** **(RQ1):** *What factors are associated with a higher presence of mental disorders among the Afro-descendant population of the Chota River basin?*

## 2. Materials and Methods

### 2.1. Sample

A cross-sectional, analytical, quantitative study was conducted in communities of the Chota River basin, comprising a sample of 557 African American individuals. The sample size was determined to ensure representativeness by age and gender, considering an expected prevalence of mental disorders of 30% (based on another local community study), a 95% confidence level, and a 5% margin of error. This yielded a minimum sample size of 323; to compensate for the design effect and ensure adequate precision, the sample size was increased.

### 2.2. Sampling Design

The sample was drawn from a population of 5118 inhabitants using two-stage cluster probability sampling in the communities of Caldera, Piquiucho, Carpuela, San Vicente de Pusir, and Pusir Grande. In the first stage, communities were randomly selected by lottery and designated as primary clusters. The second stage involved selecting zones according to the distribution established by the Ecuadorian Ministry of Public Health, which were then randomly drawn as sub-units. Within these selected zones, men and women aged 18 years or older who were habitual residents were interviewed, excluding those unable to respond to the questionnaires due to disability and lack of an accompanying person.

### 2.3. Procedure

Prior to data collection, participants were informed about the study’s objectives and scope, and written informed consent was obtained from each participant. Data collection was conducted by a team of trained researchers and students skilled in administering the instrument, who conducted individual interviews with participants in confidential settings within each community until the target sample size was reached. Risk factors such as perceived stress and experiences of abuse and maltreatment were obtained directly from the GMHAT-PC. Specific data on abuse and maltreatment experiences were extracted as dichotomous variables from the questionnaire. Other sociodemographic variables, such as economic income, were obtained through additional questions included in the semi-structured interview administered by trained interviewers.

### 2.4. Instruments

The assessment of mental disorders was conducted using the GMHAT-PC (Global Mental Health Assessment Tool—Primary Care), an instrument that identifies a wide range of mental health problems and can be used by healthcare professionals to detect and manage mental disorders in primary care among the general population. Validation studies have reported a sensitivity of 0.79 (95% CI: 0.73–0.85) and a specificity of 0.94 (95% CI: 0.83–1) [18,19,20,21]. GMHAT is used for the systematic assessment of mental symptoms and disorders, allowing for the identification of conditions such as depression, anxiety, and psychotic disorders, among others. It has been used in various settings, including inpatient services and community-based contexts [22,23]. In addition, sociodemographic variables, history of abuse, stress level, and personal and family psychiatric history were recorded.

To assess lifestyle-related factors, questions from Phase 1 of the World Health Organization (WHO) STEPS instrument were incorporated. This tool is designed for the epidemiological surveillance of behavioral risk factors associated with noncommunicable chronic diseases. The initial stage (STEPS I) gathers information on sociodemographic characteristics, tobacco and alcohol consumption, nutritional status, and physical activity. The STEPS questionnaire is widely used in population-based studies and has been validated across multiple settings, facilitating the identification of risk behaviors that impact the mental and physical health of populations [24,25].

### 2.5. Data Analysis

Data were processed and analyzed using Jamovi statistical software (version 2.6). Descriptive analysis of frequencies and percentages was conducted for sociodemographic variables and risk factors. Subsequently, prevalences of identified mental disorders were estimated, and associations were explored between independent variables (age, gender, educational level, history of violence, social support, dietary habits, physical activity) and mental health outcomes. The significance level was set at 0.05. Age was classified into three groups following standardized Latin American epidemiological conventions established by governmental health entities—young adults (18–44 years), middle-aged adults (45–64 years), and older adults (≥65 years), consistent with World Health Organization contextual recommendations for morbidity studies in developing countries.

## 3. Results

This study described sociodemographic factors and certain determinants of mental health in a population settled in northern Ecuador. It focused on data such as gender, economic status, marital status, educational level, age, and family type, as well as risk factors including history of abuse and maltreatment, stress levels, and personal and family history of mental disorders. The overall prevalence of mental disorders in the study population was 60.7% (95% CI: 56.8–64.6%), with significant differences according to gender, stress level, and economic income.

### 3.1. Sociodemographic Data

The sociodemographic characterization of the sample, consisting of 557 participants, revealed a population profile marked by social and economic vulnerability, shaping a context of significant psychosocial risk.

#### 3.1.1. Sex Distribution

Most participants were female, as shown in Table 1 (61.2%), representing greater female representation in the sample.

#### 3.1.2. Age-Group Distribution

In the age distribution, a balanced representation was found among young adults, middle-aged adults, and older adults, reflecting the demographic heterogeneity of rural African American communities.

#### 3.1.3. Marital Status

A higher proportion of single and married individuals was identified, while the categories of divorced, separated, and widowed were less represented. This distribution reflects a diversity of marital situations, with those associated with the absence of formal marital bonds or legally established stable relationships predominating.

#### 3.1.4. Education

Regarding education levels, the population exhibited predominantly low to middle educational attainment, with the greatest concentration at primary and secondary levels. The proportion of individuals with higher education was very low, likely indicating limited access to higher education levels.

#### 3.1.5. Economic Income

Marked economic precarity was determined in most of the population, with 87.8% (n = 489) earning less than the unified basic salary.

#### 3.1.6. Family Type

The most predominant family grouping was the nuclear family, although a considerable presence of monoparental households was also notable, particularly those headed by women. This family structure implies a greater economic and emotional burden, which may be associated with increased risk of depressive symptoms and anxiety in both caregivers and dependents.

To further characterize the study population, the distribution of the main risk factors evaluated—abuse and maltreatment, stress level, and personal and family psychiatric history—is presented in Table 2.

### 3.2. Risk Factors

The analysis of psychosocial risk factors and psychiatric history in the studied population revealed a complex pattern of emotional vulnerability and cumulative exposure to adverse experiences, situated within a historical context of structural marginalization.

#### 3.2.1. Abuse and Maltreatment

Although most participants did not report experiences of abuse, a considerable proportion reported histories of physical or psychological violence, with the latter being the most prevalent form (20.5%). The coexistence of physical and psychological maltreatment in some cases suggests a pattern of multifactorial violence, which may have a significant impact on emotional regulation, self-esteem, and stress coping capacity.

#### 3.2.2. Stress Level

The distribution of stress levels showed that nearly half of the participants experienced some degree of perceived stress, with a non-negligible group reporting severe stress. The predominance of mild and moderate stress may reflect community resilience mechanisms and cultural adaptation to adversity; however, the presence of severe stress in a significant percentage (7%) suggests an overflow of traditional coping strategies, which could contribute to the emergence of anxious, depressive, and somatic symptomatology.

#### 3.2.3. Personal and Family Psychiatric History

The proportion of participants with personal and family histories of mental health conditions confirmed the presence of psychobiological vulnerability in a significant segment of the population. Personal histories reflected prior exposure to depressive, anxious, or psychotic episodes. The existence of family histories suggests a hereditary or familial component that, combined with adverse environmental factors, could potentiate the clinical expression of mental disorders.

To contextualize the subsequent analysis, the distribution of the main mental disorder diagnoses identified by the GMHAT/PC in the study population is presented in Table 3.

### 3.3. Mental Disorder Prevalence

Screening using the Global Mental Health Assessment Tool (GMHAT/PC) revealed a high burden of psychiatric morbidity among the African American population of the Chota River basin. Of the 557 participants assessed, 60.7% (n = 338) presented at least one diagnosed mental disorder, predominantly affective and anxiety disorders, followed by psychotic conditions and organic disorders. Depressive disorder was the most frequent diagnosis (15.6%), constituting the primary mental health problem in the sample, while anxiety disorder ranked second at 10.2% (n = 57).

Although less prevalent, other mental health conditions were identified, including personality disorders, obsessive–compulsive disorder, phobias, and eating disorders, which broaden the observed clinical spectrum and reflect the diversity of psychopathological expressions within the community.

Bivariate analysis (showed that women exhibited higher prevalence of mental disorders (66.3%; 95% CI: 61.2–71.1) than men (51.9%; 95% CI: 45.0–58.7), with an OR of 1.82 (95% CI: 1.32–2.51; χ^2^ = 10.9, *p* = 0.001). Stress demonstrated a dose–response relationship with mental disorders—mild (OR = 2.03), moderate (OR = 4.46), and severe (OR = 27.6; global *p* < 0.001), reflecting the accumulated psychological burden in these communities. Mental disorder prevalence decreased with higher economic income (47.1% vs. 62.6%; OR = 1.87, *p* = 0.014), highlighting key socioeconomic determinants. Experiences of abuse/maltreatment significantly increased risk (physical OR = 4.15; psychological OR = 3.47; *p* < 0.001), while marital status and education showed non-significant associations (*p* > 0.05). The results of the bivariable analysis examining the association between sociodemographic factors and mental disorder prevalence are presented in Table 4.

Table 5 presents a multivariate analysis of sociodemographic factors, gender, and major psychiatric diagnoses such as depression, anxiety, and psychosis; the results revealed relevant interactions between sociodemographic variables—particularly gender and education level—with the prevalence of the main identified mental disorders. Women generally exhibited a higher proportion of psychiatric diagnoses compared to men. For major depressive disorder, a statistically significant association emerged between lower education levels and higher prevalence (*p* = 0.041), particularly among women with primary education or less. Psychosis also showed a significant link to schooling (*p* = 0.046), with the most severe cases concentrated among individuals—especially women—with lower education levels.

Anxiety disorders displayed no significant differences by education level (*p* > 0.05). Income level, however, associated significantly with anxiety (*p* < 0.01), proving more prevalent in women earning below the basic salary. This underscores the interplay between economic inequality and gender in generating psychological distress, where women in poverty experience greater emotional burden and reduced access to formal or informal support, perpetuating a cycle of vulnerability. Marital status analysis revealed a trend—though not statistically significant—of higher depression and psychosis rates among separated, divorced, or widowed women.

Table 6 presents univariate associations showing high stress and a history of abuse significantly related to higher depression risk (OR = 4.2 and 3.9, respectively; both *p* < 0.001). Stress also demonstrated statistically significant associations with anxiety (OR = 2.1, *p* = 0.016) and psychosis (OR = 1.8, *p* = 0.025).

Once the various risk factors were identified, a multivariate analysis was performed to determine which sociodemographic factors and/or risk factors are the strongest predictors of mental disorders. The results of the multivariate logistic regression analysis identifying the strongest predictors of mental disorders are presented in Table 7.

The multivariate logistic regression analysis identifies history of abuse (OR 2.44) and stress levels (OR 2.11) as the strongest independent predictors of mental disorders in this population (*p* < 0.001). Male sex was found to be a significant protective factor, associated with a 37% reduction in risk compared to females. After adjusting for these variables, socioeconomic factors such as income and educational level did not show a statistically significant association with the general diagnosis.

## 4. Discussion

Mental disorder findings reveal a troubling reality in these populations, alongside relevant associations with certain sociodemographic factors such as history of abuse and maltreatment, stress levels, gender, and economic income. The 60.7% mental disorder prevalence, obtained via the GMHAT in this community population from the Río Chota Basin, stands out as notably elevated compared to national and international studies. The U.S. survey conducted between 2001 and 2003, accounting for racial differences between whites and blacks, found that African Americans have equal or greater likelihood of presenting mental disorders. Jude Mary C.’s recent meta-analysis, including 1.3 million people of African descent, reported a depression prevalence of 20.2% (95% CI: 18.7–21.7%). U.S. studies indicate that African American adults face a 20% higher probability of experiencing serious mental health problems compared to the white population [26]. A systematic review in the World Mental Health report indicates that socially marginalized groups, including ethnic minorities, exhibit higher rates of mental disorders. However, none of these findings exceed the figure reported in the current study; the Río Chota river basin features adverse socioeconomic conditions that may amplify vulnerability to mental disorders, including high rural poverty (87.8% of the sample with incomes below basic salary), low education levels (predominance of incomplete primary/secondary schooling), and diverse family structures (51.7% nuclear but 21.2% single-parent), aligned with chronic stressors such as food insecurity, geographic isolation, and limited access to mental health services [27,28]. Another factor likely contributing to the high prevalence observed is the assessment tool used in this study. GMHAT, although validated for primary care, detects subclinical cases, enabling more frequent identification of various mental disorders. Structural racism, causing diverse social problems combined with other social determinants, generates chronic stress and trauma [29,30,31], which is important to consider as a cause of elevated prevalence.

The data also confirm the global epidemiological pattern that women present mental disorders more frequently. The explanation lies in possible greater biological and hormonal vulnerability, specific psychosocial factors, and cultural models, along with low education levels, social class, unemployment, and lack of social support that affect Afro-American women—including traditional gender roles, family caregiving overload, and greater exposure to gender violence—as suggested by studies from Galbis and Bacigalupe, and the abuse/maltreatment data from this study itself, leading to the accumulation of traumatic factors [14,32].

Most of the population was found to have incomes below the basic salary, including unemployment, and these groups exhibited the highest prevalence of mental disorders. This finding aligns with evidence from Gili’s study, which links poverty and mental health through moderate to severe psychological distress [29,33]. This association proves particularly relevant in Afro-American communities, where populations living below the poverty threshold have more than double the likelihood of reporting severe stress—alongside other conditions these groups have historically faced [27].

Regarding the spectrum of mental disorders, the study found predominance of major depressive disorder, anxiety disorder, and psychosis cases. According to the 2018 National Survey on Drug Use and Health (SAMHSA), 19.7% of Black and African American adults experienced a mental health condition in the past year [34]. In Greene et al.’s review study, 22 point prevalence studies across 12 North African countries determined that major depressive disorder ranged from 2.0% to 33.2%, with the highest prevalence in South Africa—the origin of Ecuadorian Afro-descendants. In the same study, point prevalence for anxiety disorders varied from 0.9% to 36.5% across 12 of the 36 studies reviewed [35]. The prevalence findings for depression and anxiety in this study align with Greene et al.’s review, but results differ across analyzed studies regarding psychosis data, with point prevalences ranging from 0.19% to 9.3% in just two studies from different African cities potentially reaching over 39% for lifetime prevalence. None of the studies included in Greene et al.’s review used the GMHAT/PC; however, despite diagnostic heterogeneity, they converge on DSM criteria and findings [35]. The significant presence of psychosis—alone or associated with affective disorders—requires special attention, as it may reflect both genetic vulnerabilities and the impact of chronic psychosocial stress [36]. The presence of depressive disorder, psychosis, and anxiety—in that order—showed significant correlation with higher stress levels and presence of abuse/maltreatment more than other sociodemographic variables, except low income for anxiety disorder and low education, where depressive disorder and psychosis approached statistical significance.

The data demonstrated a highly significant association between stress level and presence of mental disorders (*p* < 0.001); the progressive increase in prevalence according to stress level—reaching up to 94.9% in severe stress cases—evidences the central role of this factor in psychiatric pathophysiology. Chronic stress associates with hypothalamic–pituitary–adrenal axis dysfunction, neuroinflammation, and neuroplasticity alterations that promote the emergence of depressive and anxious symptoms [37].

Finally, the results from this study regarding maltreatment and abuse as risk factors for mental disorders revealed a pattern of high prevalence with depressive and anxious symptoms, showing a significant association (*p* < 0.001), consistent with reports by Grayson et al., where racial and structural trauma generates lasting effects on the mental health of the Afro-American community. Both physical abuse and psychological abuse act as chronic stressors that trigger mental health disorders [38]. The results of Ricks and Horan [39] found that exposure to childhood sexual abuse (40.6%) and intimate partner violence (44.9% physical, 63% emotional, 40.6% sexual) are extremely frequent and strongly associated with depressive symptoms, anxiety, and poorer quality of life, which was evidenced in the population of the Chota Valley.

The practical implications for public health of such high-prevalence findings for both anxiety and depression—and their key associated risk factors—require developing early detection programs for mental health problems in the Río Chota river basin. Additionally, they demand the implementation of interinstitutional activities focused on reducing stress, violence, and economic and educational inequities in the population, without overlooking culturally adapted strategies to address transgenerational historical trauma.

The main limitations of this study include its cross-sectional design, which prevents inferring causality; the use of screening instruments without diagnostic confirmation; and female oversampling, which may affect gender-based generalizability. Future longitudinal studies with simple random sampling and confirmed clinical diagnosis are needed to validate these associations in similar contexts.

## 5. Conclusions

The sociodemographic factors revealed a female predominance in the sample and a high proportion of young and older adults with low educational levels and high poverty rates, as most participants reported incomes below the minimum wage. This economic precariousness was significantly associated with a higher prevalence of mental disorders.

The present study confirms a high burden of psychiatric morbidity in the Afro-descendant communities of the Chota River basin, showing a significantly higher prevalence of mental disorders than the national and regional averages reported for Latin America.

The most frequent mental disorders were major depressive disorder, anxiety disorder, and psychosis with depressive symptoms, all with a female predominance.

A central role of stress and experiences of abuse/maltreatment in the genesis of mental disorders was evidenced, with a directly proportional relationship observed between the intensity of stress and the presence of mental illness (anxiety).

Physical and psychological abuse showed a 100% association with the presence of mental disorders, confirming the relevance of interpersonal trauma as a psychiatric trigger, especially depression.

## Figures and Tables

**Table 1:** Characteristics of the study population.

Variable	Frequency	Percentage of Total
Sex		
Female	341	61.2%
Male	216	38.8%
Total	557	100%
Age		
Young adults	199	35.7%
Middle-aged adults	164	29.4%
Older adults	194	34.8%
Marital status		
Married	183	32.9%
Divorced	30	5.4%
Separated	21	3.8%
Single	195	35%
Common-law union	62	11.1%
Widowed	66	11.8%
Educational level		
None	37	6.6%
Primary	264	47.7%
Secondary	208	37.3%
Higher education	48	8.6%
Economic income		
Above minimum wage	68	12.2%
Below minimum wage	489	87.8%
Family type		
Nuclear family	288	51.7%
Single-parent family	118	21.2%
Extended family	54	9.7%
Large/complex family	38	6.8%
Other	58	10.6

Own elaboration, based on data collected in communities within the Chota River basin.

**Table 2:** Presence of risk factors.

Variable	Frequency	Percentage of Total
Abuse and maltreatment		
Physical, sexual, and psychological	7	1.3%
Physical	36	6.5%
Psychological	114	20.5%
Sexual	3	0.5%
None	397	71.3%
Stress level		
Mild stress	211	37.9%
Moderate stress	93	16.7%
Severe stress	39	7.0%
No stress	214	38.4%
Personal psychiatric history		
Yes	127	22.8%
No	430	77.2%
Family psychiatric history		
Yes	71	12.7%
No	386	87.3%
Total	557	100%

Own elaboration, based on data collected in communities within the Chota River basin.

**Table 3:** Prevalences of the main mental disorder diagnoses detected by the GMHAT/PC (Global Mental Health Assessment Tool—Primary Care).

Mental Disorder	Frequencies	% of Total
Total with mental illness	338	60.7%
Total without mental illness	219	39.3
Major depressive disorder	87	15.6%
Anxiety disorder	57	10.2%
Psychosis with depression	46	8.3%
Organic mental disorder	34	6.1%
Psychotic disorder	20	3.6%
Personality disorder	19	3.4%
Obsessive–compulsive disorder	12	2.2%
Eating disorder	16	2.9%
Post-traumatic stress disorder	11	2.0%
Phobia	9	1.6%

Own elaboration, based on data collected in communities within the Chota River basin.

**Table 4:** Sociodemographic variables and risk factors in relation to the presence of mental disorders.

Variable	n	With Mental Disorder	Without Mental Disorder	% With Mental Disorder	IC 95%	OR	IC 95%OR	*p*-Value
Sex								
Female	341	226	115	66.3%	61.2–71.1	1.82	1.32–2.51	*p ≤* 0.001
Male	216	112	104	51.9%	45–58.7			
Age								*p* = 0.317
Young adults	199	113	86	56.8%	49.8–63.6	1		
Middle-aged adults	164	106	58	64.6%	57.1–71.6	1.39	0.92–2.10	
Older adults	194	119	75	61.3%	54.3–68	1.20	0.80–1.80	
Marital status								*p* = 0.073
Married	183	111	72	60.7%	53.4–67.6	1		
Divorced	30	23	7	76.7%	59.1.89.6	2.26	0.93–5.50	
Separated	21	11	10	52.4%	30.6–73.6	0.71	0.29–1.73	
Single	195	115	80	59.0%	51.8–65.9	0.93	0.62–1.40	
Common-law	62	31	31	50.0%	37.6–62.4	0.64	0.36–1.13	
Widowed	66	47	19	71.2%	59.8–80.8	1.6	0.86–2.98	
Education								*p* = 0.094
None	37	23	14	62.2%	45.9–76.2	1		
Primary	264	173	91	65.5%	59.6–71.1	1.16	0.55–2.45	
Secondary	208	119	89	57.2%	50.3–63.9	0.8	0.38–1.69	
Higher	48	23	25	47.9%	33.3–62.8	0.55	0.23–1.32	
Economic income								*p* = 0.014
Above basic salary	68	32	36	47.1%	35.3.59.1	1		
Below basic salary	489	306	183	62.6%	58.2–66.8	1.87	1.15–3.04	
Family type								*p* = 0.236
Nuclear	285	171	114	60%	54.2–65.6	1		
Single-parent	118	78	40	66.1%	57.2–74.2	1.28	0.82–2.01	
Extended	54	32	22	59.3%	45.3–72.2	0.98	0.53–1.82	
Large/extended	38	18	20	47.4%	31–64.1	0.6	0.28–1.28	
Stress level								*p* ≤ 0.001
No stress	214	96	118	44.9%	38.3–51.5	1		
Mild	211	132	79	62.6%	55.7–69	2.03	1.38–2.99	
Moderate	93	73	20	78.5%	69.4–86	4.46	2.48–8.02	
Severe	39	37	2	94.9%	82–99.4	27.6	6.39–119.2	
Abuse/maltreatment								*p* ≤ 0.001
None	325	164	161	50.5%	45–55.9	1		
Physical	36	29	7	80.6%	64.6–91.8	4.15	1.77–9.72	
Psychological	114	87	27	76.3%	67.7–83.5	3.47	2.15–5.6	
Physical, sexual and psychological	7	7	0	100%	100–100			
Sexual	3	2	1	66.7%	9.4–99.2	2.08	0.19–2.31	
Total	557	338	219	60.7%	56.8–64.6			

Own elaboration, based on data collected in communities within the Chota River basin.

**Table 5:** Multivariate analysis of sociodemographic variables, gender, and major psychiatric diagnoses.

Sociodemographic Variable	Gender	Major Depressive Disorder n (%) *p*-Value	Anxiety Disorder n (%) *p*-Value	Psychosis n (%) *p*-Value
Education Level				
Primary or less	Female	66 (35.1%)	27 (14.4%)	30 (16%)
	Male	27 (23.9%)	12 (10.6%)	9 (8%)
	*p*-value	0.041	0.46	0.046
Secondary and higher	Female	25 (16.3%)	31 (20.3%)	16 (10.4%)
	Male	19 (18.4%)	13 (12.6)	12 (11.6%)
	*p*-value	0.66		0.7
Economic Income				
Above minimum wage	Female	4 (16.7%)	3 (12.5%)	2 (8.3%)
	Male	4 (9.1%)	9 (20.5%)	2 (4.5%)
Below minimum wage	Female	83 (26.8%)	55 (17.4%)	44 (13.9%)
	Male	42 (24.9%)	16 (9.3%)	19 (11%)
	*p*-value	0.311	<0.01	0.417
Marital Status				
Married	Female	29 (24.8%)	19 (60.7%)	12 (26.1%)
	Male	13 (19.7%)	11 (39.3%)	4 (19%)
Divorced	Female	6 (33.3%)	2 (66.7%)	3 (60%)
	Male	2 (16.7%)	1 (33,3%)	2 (40%)
Separated	Female	6 (54.5%)	2 (66.7%)	2 (100%)
	Male	2 (20%)	1 (33.3%)	
Single	Female	24 (20.9%)	23 (76.7)	19 (67.9%)
	Male	17 (21.2)	7 (23.3%)	9 (32.1%)
Common-law union	Female	8 (25.8%)	8 (80%)	9 (18.4%)
	Male	8 (25.8%)	2 (20%)	3 (17.6%)
Widowed	Female	18 (36.7%)	6 (66.7%)	9 (18.4%)
	Male	4 (23.5%)	3 (33.3%)	3 (17.6%)
	*p*-value	0.299	0.801	0.3

Own elaboration, based on data collected in communities within the Chota River basin.

**Table 6:** Univariate association between risk factors and mental disorders.

Factor vs. Depressive Disorder (Ref: No Depression)	OR	IC 95%	*p* (χ^2^)
High stress	4.2	2.8–6.3	<0.001
Abuse Yes	3.9	2.6–5.9	<0.001
Factor vs. Anxiety Disorder (ref: no anxiety)	2.1	1.3–3.5	0.016
High stress	2.9	1.7–4.9	0.185
Abuse Yes	1.8	1.1–2.9	0.025
Factor vs. Psychosis (ref: no psychosis)	2.1	1.2–3.6	0.045
High stress	1.8	1.1–2.9	0.025
Abuse Yes	2.1	1.2–3.6	0.045

Own elaboration, based on data collected in communities within the Chota River basin.

**Table 7:** Determinants of general mental disorders.

Predictor Variable	OR	IC 95%	*p* (χ^2^)
Abuse	2.44	1.56–3.82	<0.001
Stress level	2.11	1.67–2.68	<0.001
Male	0.63	0.43–0.92	0.017
Low income	1.46	0.83–2.57	0.193
Education level	0.89	0.66–1.21	0.467

Own elaboration, based on data collected in communities within the Chota River basin.

## Data Availability

The data presented in this study are available on reasonable request from the corresponding author. The data is not publicly available due to privacy and ethical restrictions established by the Universidad Técnica del Norte, as they contain information that could compromise the confidentiality of study participants.

## Abbreviations

The following abbreviations are used in this manuscript:

GMHAT/PC	Global Mental Health Assessment Tool—Primary Care Version
IPAQ	International Physical Activity Questionnaire
WHO	World Health Organization
STEPS	WHO STEPwise Approach to Noncommunicable Disease Risk Factor Surveillance
UTN	Universidad Técnica del Norte
CI	Confidence Interval
OR	Odds Ratio
SDH	Social Determinants of Health
